# Modulating effects of exercise training regimen on skeletal muscle properties in female polo ponies

**DOI:** 10.1186/s12917-016-0874-6

**Published:** 2016-11-04

**Authors:** Metha Chanda, Ratchakrit Srikuea, Worakij Cherdchutam, Arthit Chairoungdua, Pawinee Piyachaturawat

**Affiliations:** 1 Department of Physiology, Faculty of Science, Mahidol University, Rama 6 Rd., Rachatewee, Bangkok, 10400 Thailand; 2 Department of Large Animal and Wildlife Clinical Science, Faculty of Veterinary Medicine, Kasetsart University Kamphaeng Saen Campus, Nakornpathom, 73140 Thailand

**Keywords:** Exercise training, Horse, Match play, Polo pony, Skeletal muscle adaptation

## Abstract

**Background:**

The match play patterns in equestrian polo are unique and require specific training programs to ensure sport performance. The effect of commonly used exercise training regimens on the adaptation of skeletal muscle is unclear. The present study investigated the modulating effects of the classic training regimen, comprised of aerobic exercise training with increasing exercise intensities and varying duration combined with match play, on the properties of muscle in polo ponies. Nine healthy adult female polo ponies were subjected to four consecutive subsets of 1 year classic training regimen including basal activity (B), low intensity (L), low to moderate intensity (LM), and low to moderate intensity training plus match play during polo tournament (LMP), respectively. At the end of each training period, gluteus medius muscle samples were taken for determination of muscle fiber type distribution, muscle metabolic capacity, capillary density, and lipid and glycogen content. The expression profile of metabolic genes including succinate dehydrogenase (SDH), phosphofructokinase (PFK), glycogen phosphorylase (PYG), and glycogen synthase (GYS) were also measured.

**Results:**

Among all exercise training subsets, only LMP exercise period caused an increase in the number of oxidative fibers (type IIa), along with increases in properties related to oxidative metabolism including high capillary density, intramuscular lipid content, and expression of SDH and PYG genes, with a corresponding decrease in the number of type IIx muscle fibers.

**Conclusion:**

The combination of low to moderate and high intensity training in LMP are only sufficient to induce changes in oxidative characteristics. As the first scientific evidence providing such insight about the classic polo training regimen, the data forms a basis for further consideration in training program design.

**Electronic supplementary material:**

The online version of this article (doi:10.1186/s12917-016-0874-6) contains supplementary material, which is available to authorized users.

## Background

Achievement in competition of athletic horses depends on genetic contributions and the ability to improve muscle performance via exercise training. The effectiveness of the response to the training depends upon the extent of stimuli [1], training volume, as determined by frequency, intensity, and duration [2]. Adaptation of muscle properties to exercise training is well documented in several equestrian disciplines including racing [2, 3], endurance riding [4], and show jumping [5]. Aerobic exercise training is generally included in basic exercise training programs in all equestrian horses inducing adaptation of skeletal muscle towards oxidative characteristics [2, 6, 7]. Increases in the proportion of oxidative muscle fibers (type IIa), number of capillaries, and SDH activity have been demonstrated after aerobic exercise training in Thoroughbred horses [2]. Moreover, aerobic training reportedly increases activity of 3-hydoxyacyl-CoA dehydrogenase (HAD) and citrate synthase (CS) accompanied by decreased glycogen utilization in Standardbred horses [6]. At a cellular level, exercise training effects several genes responsible for oxidative energy metabolism and muscle structure. Training increases expression of cytochrome c oxidase subunit 4 (COX4), pyruvate dehydrogenase complex (PDC), and pyruvate dehydrogenase kinase (PDK) in Thoroughbred horse [8, 9]. In contrast, anaerobic exercise training promotes glycolytic capacity in some sport disciplines, i.e. Thoroughbred racehorses [10].

Although the majority of horses in equestrian sports undergo individual exercise training and competition, the difference in exercise regimen is notable in equestrian polo. Polo ponies are trained as a group during the preparation period before tournaments. In tournaments, two teams of four players play against each other on a grass field area of approximately ten acres [11]. A game is made up of 4–6 periods (chukkas) 7 min in duration each. The common training regimen in equestrian polo, consists of three phases of increasing intensity, starting with low to moderate intensity – walking and trotting – and progressing to high intensity — particular field training for specific activities and match play [12]. During polo games, the horses undergo intermittent high intensity exercise as well as swift changes in speed and direction. Hence, both muscle oxidative and glycolytic capacities are crucial factors for accomplishment during polo match play in seasonal tournaments. Unfortunately, there is no evidence of the effectiveness of the classic exercise training regimen commonly used in polo ponies on their skeletal muscle properties. Therefore, the present study investigated the effects of the polo training regimen on the skeletal muscle properties related to the muscular performance including fiber type composition, metabolic capacity, substrate utilization, and the expression of metabolic genes in polo ponies.

## Methods

### Horses

Nine healthy polo ponies (mares, age 11.9 ± 2 years, weight 452.6 ± 14.8 kg) from a Thai polo and equestrian club (Pattaya, Chonburi, Thailand) were recruited. All horses regularly participated in polo seasonal tournaments (5 months/year). They were housed in 3 × 3 m^2^ separate stalls, fed with standard commercial pellet twice daily, and had free access to water and pangola hay.

### Exercise training program and muscle sampling

All horses were subjected to a training program with progressive increases in exercise intensity. Horses were randomly assigned to participate in 0–14 goal match play during polo tournament. The exercise program consisted of four consecutive periods of different exercise intensities including basal activity (B); low intensity (L); low to moderate intensity exercise (LM); and low to moderate intensity plus match play (LMP). The LMP period was divided into low to moderate intensity exercise (LM), training match play (TM), and official match play (OM). The activities, mode, frequency, and duration of each training period are summarized in Table 1. Exercise intensity was determined by heart rate (HR), expressed as a percent of maximum heart rate (%HR_max_) based on the regression analysis [13]. The duration of training period, speed, distance covered, and HR were recorded using Polar RC3 GPS sport watches connected to H3 heart rate sensors (Polar Electro, Kempele, Finland). Blood lactate concentrations were determined before and immediately after exercise sessions and match play using an Accutrend® Plus system autoanalyzer (Roche Diagnostics, Rotkreuz, Switzerland). During training match or official match play, all parameters were recorded from the horses ridden by the player at position number 2 (attack) for four chukkas.

**Table 1:** Practical classic exercise training regimen for one year

Training phase	Training period	Exercise mode	Exercise frequency	Exercise duration (estimated min)	Exercise intensity
1. Basal activity (B)	1^st^ -22^nd^ wk	Resting in stable and pasturing in paddock during off-season	Graze in the paddock 2–3 h a day	-	-
2. Low intensity exercise training (L)	23^th^-26^th^ wk	Walking only	Twice a day, 6 days/week	~60	Very low
3. Low to moderate intensity exercise training (LM)	27^th^-30^th^ wk	Walking (25 min) **→** Trotting (15min) **→** Walking (20 min)	Twice a day, 6 days/week	~60	Low to Moderate
4. Low to moderate intensity exercise training plus match play (LMP)	31^st^-53^rd^ wk	Walking (25 min) **→** Trotting (15min) **→** Walking (20 min) (LM session)	Twice a day or once a day on the day with competition. 6 days/week	~60	Low to Moderate
		Training (TM) or official (OM) match play sessions	A horse plays 2–4 chukkas a day, 3–4 days/week	~7	Moderate to Very hard

On competition days in LMP period (polo tournaments), horses trained at low to moderate intensity exercise (LM session) in the morning and participated in polo match play in the evening 3–4 day/week

Twenty-four hours after each training session, gluteus medius samples were taken by microbiopsy according to the modified method of Lindholm and Piehl [14]. Briefly, skin was subcutaneously injected with local anesthesia (1 ml of 2 % lidocaine) (L.B.S. Laboratory, Bangkok, Thailand). Then, muscle samples were taken at the middle of an imaginary line from tuber coxae to the greater trochanter of femoral bone at 50 mm depth using a 14 G semi-automated microbiopsy needle (SuperCore™, Argon Medical Devices, Texas, USA). The biopsy depth used has been employed in previous studies to collect mixed types of muscle fibers [15–17]. Muscle samples (wet weight approximately 60–70 mg) were divided into two portions. A piece of each sample was immediately embedded with optimal cutting temperature (O.C.T.) compound (Electron Microscopy Sciences, Pennsylvania, USA), then frozen in melting isopentane (Sigma-Aldrich, Missouri, USA), and stored at −80 °C for histochemical/immunohistochemical analyses. The other muscle portion was directly frozen in liquid nitrogen and stored at −80 °C for molecular analyses.

### Histochemical and immunohistochemical analyses

Transverse cross-sections of O.C.T. preserved samples were obtained using cryostat (Leica Biosystems, Nussloch, Germany) at −20 °C. Five μm thickness serial sections were stained for succinate dehydrogenase (SDH) level, fiber type composition, and capillary density evaluations. The intramuscular lipid and glycogen contents were also stained using ten μm thickness serial sections. All protocols were performed at room temperature unless noted.

### Fiber type composition

Muscle fiber types were determined according to the modified method of Kawai et al. [18]. Sections were air dried for 10 min and pre-incubated in 1 % normal goat serum (Sigma-Aldrich, Missouri, USA) for 10 min. Thereafter, three different primary monoclonal antibodies were applied separately including: 1) fast myosin (Sigma-Aldrich, Missouri, USA) (1:4000) that specifically binds with myosin heavy chain (MHC) IIa and IIx; 2) SC-71 (Developmental Studies Hybridoma Bank, Iowa, USA) (1:1000) that specifically binds with MHC-IIa; and 3) BF-35 (Developmental Studies Hybridoma Bank, Iowa, USA) (1:1000) that reacts with all MHCs except MHC-IIx. The serial sections were then incubated overnight in a moist chamber. After washing with PBS, the sections were incubated with goat anti-mouse IgG conjugated with horseradish peroxidase (HRP) secondary antibody (Merck Millipore, Massachusetts, USA) (1:500) for 2 h and washed with PBS. Subsequently, sections were incubated with diaminobenzidine tetrahydrochloride (DAB) (Sigma-Aldrich, Missouri, USA) in a dark environment for 3 min and mounted with temporary mounting medium (Vectors Lab, California, USA).

### Muscle oxidative capacity

Oxidative fibers were determined by SDH staining according to the method previously described [19]. Briefly, muscle sections were incubated at 37 °C in solution containing 0.2 M PBS, succinic acid disodium, and nitroblue tetrazolium (Sigma-Aldrich, Missouri, USA) in a dark environment for 1 h. Thereafter, the sections were rinsed with series of acetone (30, 60, and 30 % concentrations) followed by distilled water prior to mounting with temporary mounting medium (Vectors Lab, California, USA).

### Intramuscular lipid and glycogen content

Lipid content was determined using Oil Red O staining according to the method of Lillie and Ashburn [20]. Glycogen content was evaluated by using Periodic Acid Schiff (PAS) staining according to the modified method of Kocsis et al. [21].

### Capillary density

The number of capillaries were analyzed using double labeling of anti-endothelial cell (CD31) and dystrophin protein to visualize muscle fiber. Briefly, the sections were pre-fixed with ice-cold acetone at 4 °C for 10 min, then rehydrated with PBS. After blocking with 10 % normal goat serum (Sigma-Aldrich, Missouri, USA) for 1 h, the sections were simultaneously incubated with polyclonal rabbit anti-CD31 primary antibody (Abcam, Cambridge, UK) (1:100) and monoclonal mouse anti-dystrophin primary antibody (Sigma-Aldrich, Missouri, USA) (1:100) for 1 h, and then washed with PBS. Thereafter, Alexa Fluor 568 conjugated-goat anti-mouse secondary antibody (Invitrogen, California, USA) (1:500) and Alexa Fluor 488 conjugated-goat anti-rabbit secondary antibody (Invitrogen, California, USA) (1:500) were applied simultaneously for 1 h. After washing with PBS, the sections were post-fixed with 4 % paraformaldehyde for 10 min and counterstained with DAPI (Invitrogen, California, USA) (1:10,000) for 5 min. The stained sections were mounted with fluoroshield mounting medium (Sigma-Aldrich, Missouri, USA).

### Image analysis

All stained sections were visualized under a microscope (Olympus BX53) connected with a digital camera (Olympus DP73) (Olympus, Tokyo, Japan) and captured at 200× magnification, except fiber type composition determination that were captured at 100× magnification. All histological images were quantified using ImageJ software version 1.47v. Histological images of SDH, Oil Red O, and PAS stained sections were analyzed as the area of expression according to the modified method of Rinnankoski-Tuikka et al. [22]. A minimum of four captured images were analyzed for each staining. The color images were converted to 8-bit gray-scale (range of grey levels 0–255) images before processing. The threshold of staining was manually adjusted and kept constant between samples. The oxidative capacity, intramuscular lipid, and glycogen contents were expressed as percentage of the measured area. Muscle fiber types were classified according to the specific reaction with antibodies as type I, IIa, IIa/x, and IIx. The percentage of fiber distribution and cross-sectional area were measured and calculated from between 300–500 muscle fibers. The capillary density was quantified as capillary per fiber ratio from 150 to 250 muscle fibers.

### Quantitative real-time PCR for mRNA expression

Total RNAs were extracted from muscle samples using a Trizol® Reagent (Ambion, Texas, USA) and enhancing precipitation with Rnase-free glycogen (Ambion, Texas, USA) according to the manufacturer’s instructions. The purity and yield of total RNA were determined by measuring the absorbance at 260 and 280 nm using a NanoDrop 2000c UV–vis spectrophotometer. cDNAs were synthesized by using iScript™ cDNA synthesis kit (Bio-Rad, California, USA) and stored at −20 °C until analysis. The cDNA products were analyzed by real-time PCR using KAPA SYBR® FAST quantitative PCR kit (Kapa Biosystems, Massachusetts, USA) in StepOne Real Time PCR system (Applied Biosystems, Massachusetts, USA). The followings are sequences of the target gene primers: SDH (NM_001163823), 5’-GGCTCTACGAGTGCATCCTC-3’ and 5’-GCCCAAATACTTGTCCCCGT-3’; PFK (NM_001081922), 5’-TCCAGCCAGCTTCCTCAATTCTG-3’ and 5’-CATCCAGGGCAACCGAGTG-3’; GYS (NM_001126125), 5’-GCTTTGGGACACCTGCAACA-3’ and 5’-GCCAGGAACTCACCCAGGAAC-3’; PYG (NM_001145253), 5’-TCGACAGATCATCGAGCAGC-3’ and 5’-CTTTAAACCGGTCGTGGTGC-3’; β-actin (NM_001081838), 5’-GCACCAGGGCGTGATGG-3’ and 5’-TCGATGGGGTACTTGAGGGT-3’. The mRNA level of each gene was normalized to mRNA level of β-actin.

### Statistical analysis

The data from histological analysis and metabolic enzyme mRNA expression were analyzed using one-way ANOVA with repeated measurement followed by Tukey’s post hoc. Normal distribution and homogeneity of variance were analyzed by Kolmogorov Smirnov test and Levene’s test, respectively. Paired Student’s t-test was used where applicable. The statistical analysis was performed using SPSS version 18.0.0. All data are expressed as means ± SD. Significant differences were considered at *p* < 0.05.

## Results

### Heart rate and blood lactate concentration

Heart rate and blood lactate concentration in relation to distance covered and speed during each exercise training period of the classic polo training regimen are shown in Table 2. The maximum heart rate during exercise in B period was 37.1 ± 1 bpm (17 ± 1.1 % HR_max_) and 41.7 ± 2.9 bpm (19 ± 1.3 % HR_max_) during exercise in L period. The maximum heart rate increased substantially during LM period (HR = 144.3 ± 20.5 bpm, 65.6 ± 6.2 % HR_max_) (*p* < 0.01). When horses participated in a polo match play, maximum heart rate increased substantially to approximately 175.0 ± 5.2 bpm (80 ± 1.7 % HR_max_) in TM sessions and 195.3 ± 13.1 bpm (88.8 ± 6.7 % HR_max_) with a maximum heart rate of 208 bpm during OM sessions. Of note, exercise in both L and LM periods produced blood lactate below threshold (<4 mmol/L); while, exercise in both kinds of match play (TM and OM) sessions produced blood lactate above threshold (>4 mmol/L). Furthermore, lactate concentration during OM was considerably higher than during TM (OM; 8.9 ± 0.6 mmol/L vs. TM; 4.2 ± 0.8 mmol/L). Maximum heart rate variation corresponded to exercise intensity – low, moderate, and moderate to very hard for L, LM, and LMP, respectively.

**Table 2:** Heart rate, blood lactate, speed, distance, and duration of a single bout of exercise in each exercise training period

Parameters		Consecutive exercise training periods for 1 year					
		B (22 weeks)	L (4 weeks)	LM (4 weeks)	LMP (22 weeks)		
					LM session	M	
						TM session	OM session
Distance covered (km)		-	6.6 ± 0.2	7.3 ± 0.1	7.3 ± 0.3	2.8 ± 0.1	1.4 ± 0.01
Duration (min)		-	58.3 ± 1.2	60.3 ± 0.6	60.1 ± 0.5	11.3 ± 0.8	7.9 ± 0.3
Speed (km/h)	Maximum speed	-	-	16.9 ± 1.1	17.0 ± 0.7	37.1 ± 5.2	47.4 ± 2.9
	Average speed	-	6.7 ± 0.1	6.7 ± 0.7	6.7 ± 0.8	14.7 ± 1.2^£**†**^	10.4 ± 0.4^£† ¶^
HR (bpm)	Resting HR	37.1 ± 1	37.3 ± 1.3	36.0 ± 2.5	36.7 ± 1.6	38.0 ± 2.0	42.3 ± 3.5
	Maximum HR	-	41.7 ± 2.9	144.3 ± 20.5	147.3 ± 13.3	175.0 ± 5.2	195.3 ± 13.1
	Average HR	-	38.0 ± 4	72.0 ± 4*	68.3 ± 4.4	132.3 ± 4.5^£ **†**^	137.0 ± 5.0^£ †^
	% HR_max_	17 ± 1.1	19 ± 1.3	65.6 ± 6.2	66.8 ± 4.1	80 ± 1.7^£ **†**^	88.8 ± 6.7^£ **†**^
Blood lactate (mmol/L)	Before exercise	-	<0.9	1.4 ± 0.3	1.3 ± 0.2	1.9 ± 0.1	1.2 ± 0.1
	Immediately after exercise	<0.9	<0.9	1.1 ± 0.1	1.1 ± 0.1	4.2 ± 0.8^**†**^	8.9 ± 0.6^† ¶^

The activities in each period are different in the exercise intensity (means ± SD) (*n* = 4)

*B* basal activity, *L* low intensity exercise, *LM* low to moderate intensity exercise, *LMP* low to moderate intensity exercise plus match play, *M* match play, *TM* training match, *OM* official match, *HR* Heart rate

^£^ *p* < 0.01; significantly different from L period

^†^ *p* < 0.01; significantly different from LM

^¶^ *p* < 0.01; significantly different from TM

^*^ *p* < 0.01; significantly different from L period

### Muscle fiber type composition and fiber size

**Fig. 1:**
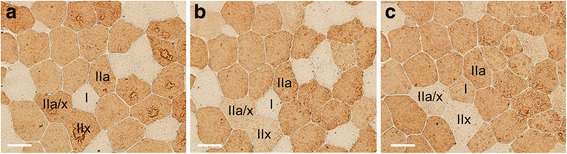
Immunohistochemical staining of transverse sections of gluteus medius muscle from polo ponies at L period with monoclonal antibody against (**a**) MHC IIa, and IIx (fast myosin), (**b**) MHC IIa (SC-71), and (**c**) MHC all but IIx (BF-35). Muscle fibers are identified as type I (*unstained* in **a** and **b**), type IIa (*dark brown* stain in **b**) type IIa/x (*light stain* in **b** and **c**), and type IIx (*unstained* in **c**). Scale bars = 50 μm

**Fig. 2:**
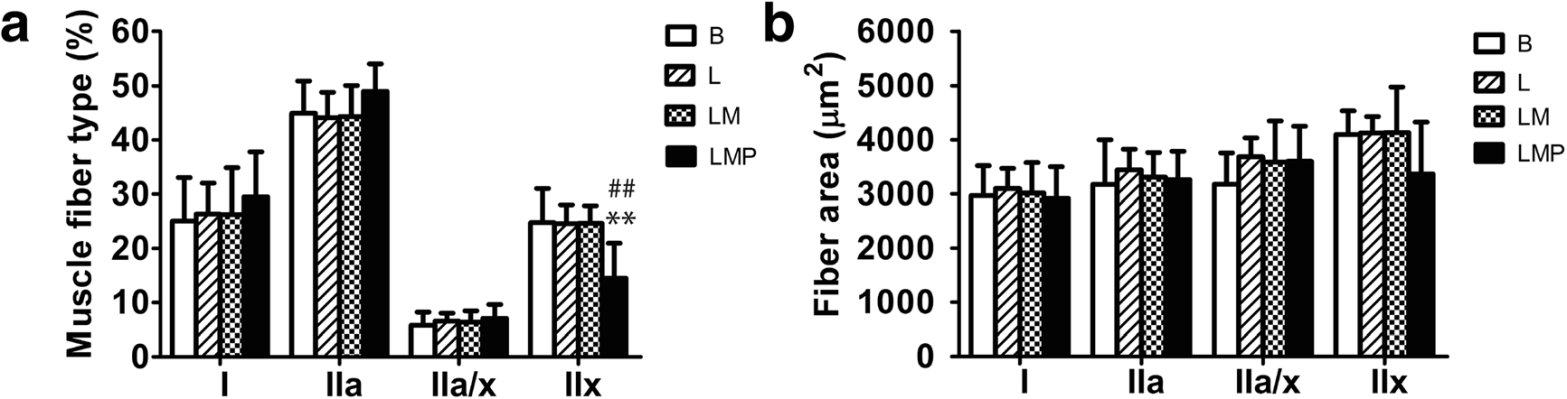
Fiber type distribution and cross sectional area (CSA) (μm^2^) of fibers in response to different exercise periods in polo ponies. **a** Changes in percentage of each muscle fiber type after basal activity (B), low intensity exercise training (L), low to moderate intensity exercise training (LM), and low to moderate intensity exercise training plus match play (LMP) periods. **b** Muscle CSA in response to the exercise regimen after B, L, LM, LMP periods. ** *p* < 0.01, significantly different from B value; ^##^ *p* < 0.01, significantly different from L value

As shown in Fig. 1, muscle fiber types were differentially stained for specific expression of myosin heavy chain (MHC) isoforms. In the basal period (B), gluteus medius muscle consisted of type I, type IIa, type IIa/x, and type IIx at approximately 25.1 ± 8.0, 44.9 ± 6.0, 5.8 ± 2.4, and 24.8 ± 6.3 %, respectively. The percentage of muscle fiber type IIx significantly decreased in LMP compared to B and L periods (*p* < 0.01) (Fig. 2a). The decrease in the number of type IIx was accompanied by an increase in the number of type IIa and I muscle fibers, but this finding was not statistically significant (*p* > 0.05). Analysis of changes of fiber type in individual animals revealed 8 out of 9 animals experienced increases in type IIa fibers, and 7 out of 9 animals experienced increases in type I fibers after the LMP period compared to after the L period*.* Additional details are provided in the Additional file 1. The cross-sectional area (CSA) (μm^2^) for muscle fiber types I, IIa, IIa/x, and IIx, were 2971 ± 549.5, 3176 ± 822.7, 3177 ± 578.9, and 4100 ± 434.2 μm^2^ respectively. No statistically significant differences in CSA of individual muscle fiber types were found throughout the course of the study (Fig. 2b).

### Oxidative capacity, substrate contents, and capillary density

**Fig. 3:**
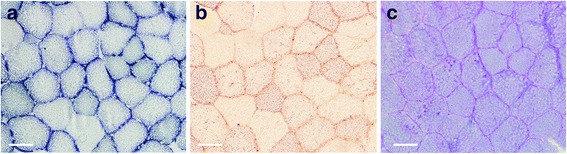
Histochemical staining of gluteus medius muscle from polo ponies at L period. **a** SDH staining shows the *dark-purple* color that marks oxidative enzyme activity. **b** *Oil red* O staining shows the lipid droplet (*red color*) within the muscle fiber. **c** PAS Schiff reagent staining shows the *red-purple* color of the glycogen rich fiber. Scale bars = 50 μm

**Fig. 4:**
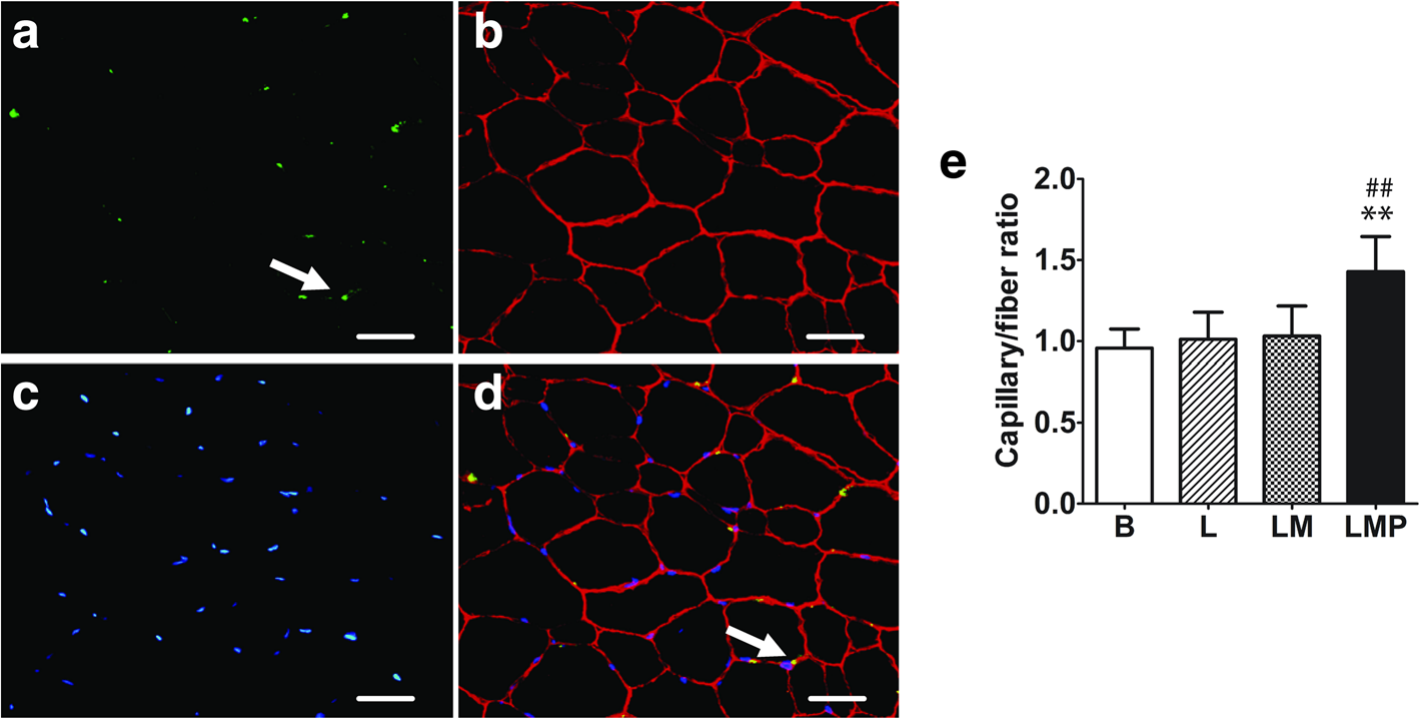
Number of capillary density in response to different exercise regimens in polo pony including basal activity (B), low intensity exercise training (L), low to moderate intensity exercise training (LM), and low to moderate intensity exercise training plus match play (LMP) periods. **a** Immunostaining of CD31 to illustrate number of capillaries, (**b**) dystrophin protein visualization of muscle fiber membrane structure, (**c**) counterstaining with DAPI for nuclear localization and (**d**) merged image. *White arrows* indicate the capillary (*green spot*) within the muscle fiber. Scale bars = 50 μm. **e** Capillary density expressed as capillary per fiber ratio. Scale bars = 50 μm. ** *p* < 0.01, significantly different from B value; ^##^ *p* < 0.01, significantly different from L value

Histological images of SDH staining, as well as intramuscular lipid and glycogen contents are shown in Fig. 3. The number of SDH-positive fibers significantly increased after the polo tournament period (LMP) compared to B and L periods (B; 35.9 ± 11.2 % vs. LMP; 74.4 ± 4.7 %, *p* < 0.01 and L; 36.9 ± 8.6 % vs. LMP; 74.4 ± 4.7 %, *p* < 0.01). Intramuscular lipid content also increased significantly after LMP period (B; 4.0 ± 0.8 % vs. LMP; 7.8 ± 2.0 %, *p* < 0.01 and L; 4.2 ± 0.2 % vs. LMP; 7.8 ± 2.0 %, *p* < 0.01); whereas, glycogen content was not altered throughout the study (Table 3). The capillary density expressed as capillary per fiber ratio significantly increased after LMP period (B; 1.0 ± 0.1 % vs. LMP; 1.4 ± 0.2 %, *p* < 0.01 and L; 1.0 ± 0.2 % vs. LMP; 1.4 ± 0.2 %, *p* < 0.01) (Fig. 4).

**Table 3:** Oxidative (SDH) capacity, intramuscular lipid content, and glycogen content (% area of expression) after basal activity (B), low intensity exercise training (L), low to moderate intensity exercise training (LM), and low to moderate intensity exercise training plus match play (LMP) periods

Metabolic properties	Consecutive exercise training activities			
	B	L	LM	LMP
Oxidative (SDH) capacity	35.9 ± 11.2	36.9 ± 8.6	46.1 ± 6.0	74.4 ± 4.7**^,##^
Lipid content	4.0 ± 0.8	4.1 ± 0.2	4.5 ± 0.9	7.8 ± 2.0**^,##^
Glycogen content	30.7 ± 4.7	30.2 ± 1.7	30.5 ± 2.8	29.9 ± 3.2

Each values were expressed as Mean ± SD (*n* = 9)

**p < 0.01, significantly different from B value

^##^ *p* < 0.01, significantly different from L value

### Expression of metabolic genes

**Fig. 5:**
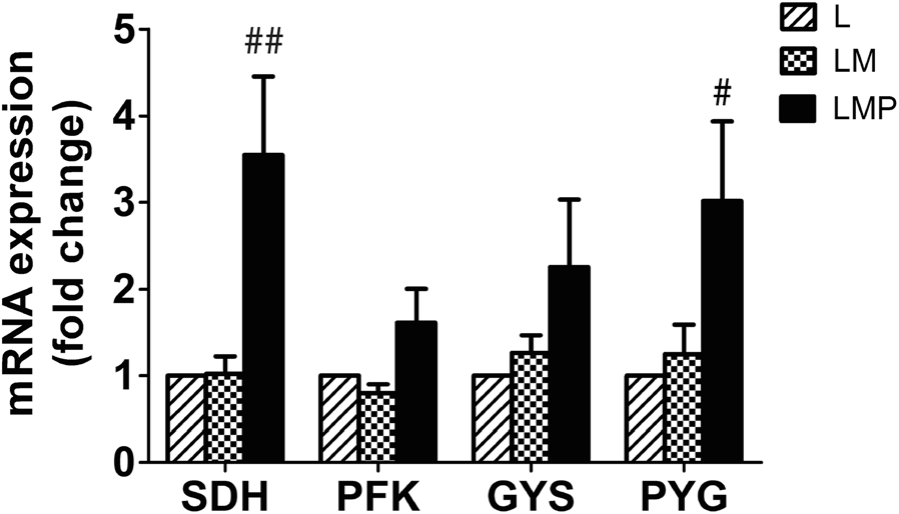
Alterations in gene expression related to different exercise periods in polo ponies. Fold changes of metabolic enzyme mRNA expression including succinate dehydrogenase (SDH), phosphofructokinase (PFK), glycogen synthase (GYS), and glycogen phosphorylase (PYG) after basal activity (B), low intensity exercise training (L), low to moderate intensity exercise training (LM), and low to moderate intensity exercise training plus match play (LMP)periods. ^##^ *p* < 0.01, significantly different from L value; ^#^ *p* < 0.05, significantly different from L value

As the exercise intensity and the histological results of basal activity (B) and low intensity exercise training (L) period were similar, mRNA expression analysis at B period was omitted. After the polo tournaments (LMP period), expression of SDH and PYG genes increased significantly (~3.5 fold, *p* < 0.01, and ~3 fold, *p* < 0.05, respectively); whereas, there was a tendency to increase expression of PFK and GYS genes (~1.6 fold, *p* = 0.076, and ~2.3 fold, *p* = 0.072, respectively) (Fig. 5).

## Discussion

According to the unique pattern of competition in equestrian polo, the properties of skeletal muscle are crucial factors determining performance in official match play. Surprisingly, little is known about the effects of the classic exercise regimen in equestrian polo on the adaptation of skeletal muscle properties and their relationship to the specific physiological demands of the sport. Interestingly, the major finding in the present study showed the exercise training regimen, particularly in LPM period of equestrian polo involving high intensity activity, evoked adaptation towards oxidative characteristics rather than glycolytic properties. Additionally, the training volume of the other training phases (L and LM) in the classic training regimen were not sufficient to induce changes in muscle properties. The oxidative adaptation of muscle fibers in the present study was evident in both biochemical and histological alterations. Muscle fiber types transitioned from fast to slow MHC phenotypes together with increased capillary density, oxidative enzymes, and associated substrates. The findings represent the first report on muscle adaptation in response to the classic exercise regimen in polo ponies.

Percent of maximal heart rate (%HR_max_) has been used as an alternative to percent of VO_2max_ to indicate exercise intensity [13, 23, 24]. In the present study, exercise intensity was estimated from %HR_max_ and was classified as follows: very low (<50 % HR_max_), low (50–63 % HR_max_), moderate (64–76 % HR_max_), hard (77–93 % HR_max_), and very hard (≥94 % HR_max_) as previously described [23]. Exercise intensity in L and LM were considered very light (L: 17 %%HR_max_; LM: 33%HR_max_), with a higher average heart rate during exercise in LM period reflecting the slightly higher intensity of exercise compared to L period. When horses participated in polo match play ,~7 min/chukka, they spent ~1 min ridden above 90 % HR_max_ (HR ≈ 195–208 bpm), followed by ~3 min ridden within ~68–89 % HR_max_ (HR ≈ 150–195 bpm), and another ~3 min ridden within ~41–68 % HR_max_ (HR ≈ 90–150 bpm). Thus, the majority of exercise period during match play in LMP period was considered moderate to very hard. The classical exercise pattern in equestrian polo utilizes both aerobic and anaerobic energy [24]. The latter is commonly indicated by blood lactate accumulation [25, 26]. In the present study, after low intensity (L) and low to moderated intensity (LM) exercise periods, blood lactate concentrations were far below the anaerobic threshold (<4 mmol/L), indicating these training regimens rely mainly on aerobic energy metabolism. However, it is worth noting that both intermittent high intensity exercise training match play (TM) and official match play (MO) in the LMP produced blood lactate concentrations above the anaerobic threshold (>4 mmol/L), indicating the anaerobic energy pathway becomes involved during polo match play. Accordingly, muscle glycolytic capacity is also crucial for this sport; however, classic exercise training regimens promotes the muscle oxidative capacity in all polo ponies rather than anaerobic properties found in only a few horses (Fig. 5c). The disparity between high glycolytic capacity required in match play and a training program promoting oxidative capacity only does not promote the sport capability of polo ponies. As glycolytic capacity is enhanced by anaerobic exercise training, the question arises as to whether additional anaerobic exercise –such as sprint exercise training – could increase glycolytic capacity, in turn, improving sport performance.

The effect of aerobic exercise training on muscle fiber type composition in horses has been reported [27, 28]. Transitions of MHC from type IIx to IIa during early training phases at approximately 3 months were observed [28]. As training duration increased up to 8 months, further muscle fiber transitions from MHC IIa to I were noted [20]. The changes reflected a dose–response relationship between exercise duration and magnitude of muscle property changes [21]. In the present study, type IIx fibers decreased along with marginal increases in type IIa muscle fibers in the post-LMP period. Analysis of fiber type changes in individual animals revealed eight out of nine animals showed increases in type IIa fibers, and seven out of nine animals showed increases in type I fibers after LMP compared to after L period. Thus, exercise intensity, duration, and the time course for sample collection are the primary variables accounting for the present results. As muscle samples were obtained at 1 month (L period), 2 months (LM period), and 7 months (LMP period) post-exercise onset (B period); it is possible insufficient exercise volume after 1 and 2 months of exercise training explains the lack of observable changes in fiber composition. Samples to represent LMP period were collected after 7 months of training; therefore, muscle fiber transitions from MHC IIa to I may be partially attributed to training duration instead of mode or phase; however, there was only a marginal increase in MHC IIa and a very slight increase in MHC I at the end of LMP period (Fig. 2a).

Increased oxidative capacity, as evidenced by SDH-positive staining, was concomitant with increased capillary density in polo ponies as has been reported in studies of angiogenesis [29]. Increased blood flow to muscle leads to splitting of capillaries as well as extensive endothelial cell proliferation and the formation of microtubes [30]. Thus, the classic exercise training regimen promotes oxidative properties in skeletal muscle of polo ponies.

In addition, expression of metabolic gene mRNA was consistent with phenotypic adaptation to training in polo ponies. At the end of polo tournaments, increased expression of oxidative marker (SDH) gene was closely correlated with increased numbers of SDH positive-stained fibers, indicating the classic exercise regimen induced oxidative capacity adaptation at a cellular level. Also, as anaerobic glycolysis exclusively uses carbohydrates in the absence of oxygen, increases in PYG along with marginal elevations of GYS and PFK mRNA expression were consistent with the increase in anaerobic glycolysis during polo tournaments. The classic exercise training regimen could increase, to a lesser extent than oxidative characteristics, glycolytic properties in muscle of polo ponies.

Since there were no changes to muscle properties after LM training period in the classic exercise regimen, polo ponies may start participating in polo tournaments with insufficient sport-specific performance. As a result, polo ponies are at potential risk of physical injury and metabolic disorders – such as exertional rhabdomyolysis – during and after polo match play. To improve muscle performance and minimize injury risk, starting exercise training further in advance of competition, as well as incorporating more intensity and longer duration sessions may be useful for the improvement of muscle performance.

## Conclusion

The classic exercise regimen in polo ponies profoundly enhances oxidative capacity of muscle. To a lesser extent, it had a tendency to elevate glycolytic capacity. These results provide beneficial information for future research on the development of training programs to improve specific muscle performance in equestrian polo and associated equestrian sports.

### Section

Clinical pathology, physiology and immunology.

## Additional file

Additional file 1: Table S1. Muscle fiber type I obtained after different periods of exercise training and activities. (DOCX 20 kb)

## Abbreviations

B: Basal activity
CSA: Cross-sectional area
DAB: Diaminobenzidine tetrahydrochloride
DAPI: 4’,6-diamidino-2-phenylindole
GYS: Glycogen synthase
L: Low intensity exercise
LM: Low to moderate intensity exercise
LMP: Low to moderate intensity exercise plus match play
MHC: Myosin heavy chain
O.C.T.: Optimal cutting temperature
OM: Official match play
P: Phosphate buffer saline
PAS: Periodic acid–Schiff
PFK: Phosphofructokinase
PYG: Glycogen phosphorylase
SD: Standard deviation
SDH: Succinate dehydrogenase
SPSS: Statistical package for the social sciences
TM: Training match play

## References

[1] Tyler CM, Golland LC, Evans DL, Hodgson DR, Rose RJ (1998). Skeletal muscle adaptations to prolonged training, overtraining and detraining in horses. Pflugers Arch.

[2] Rivero JL, Ruz A, Marti-Korff S, Estepa JC, Aguilera-Tejero E, Werkman J, Sobotta M, Lindner A (2007). Effects of intensity and duration of exercise on muscular responses to training of thoroughbred racehorses. J Appl Physiol (1985).

[3] Miyata H, Sugiura T, Kai M, Hiraga A, Tokuriki M (1999). Muscle adaptation of Thoroughbred racehorses trained on a flat or sloped track. Am J Vet Res.

[4] Rivero J, Ruz MC, Serrano A, Diz A (1995). Effects of a 3 month endurance training programme on skeletal muscle histochemistry in Andalusian, Arabian and Anglo‐Arabian horses. Equine Vet J.

[5] Rietbroek NJ, Dingboom EG, Joosten BJ, Eizema K, Everts ME (2007). Effect of show jumping training on the development of locomotory muscle in young horses. Am J Vet Res.

[6] Hodgson DR, Rose RJ, DiMauro J, Allen JR (1985). Effects of a submaximal treadmill training programme on histochemical properties, enzyme activities and glycogen utilisation of skeletal muscle in the horse. Equine Vet J.

[7] Eaton MD, Hodgson DR, Evans DL, Rose RJ (1999). Effects of low- and moderate-intensity training on metabolic responses to exercise in thoroughbreds. Equine Vet J Suppl.

[8] Eivers SS, McGivney BA, Fonseca RG, MacHugh DE, Menson K, Park SD, Rivero J-LL, Taylor CT, Katz LM, Hill EW (2010). Alterations in oxidative gene expression in equine skeletal muscle following exercise and training. Physiol Genomics.

[9] McGivney BA, McGettigan PA, Browne JA, Evans AC, Fonseca RG, Loftus BJ, Lohan A, MacHugh DE, Murphy BA, Katz LM (2010). Characterization of the equine skeletal muscle transcriptome identifies novel functional responses to exercise training. BMC Genomics.

[10] Eto D, Yamano S, Mukai K, Sugiura T, Nasu T, Tokuriki M, Miyata H (2004). Effect of high intensity training on anaerobic capacity of middle gluteal muscle in Thoroughbred horses. Res Vet Sci.

[11] Ferraz GC, Soares OA, Foz NS, Pereira MC, Queiroz-Neto A (2010). The workload and plasma ion concentration in a training match session of high-goal (elite) polo ponies. Equine Vet J Suppl.

[12] Giraudet A, Hinchcliff KW, Kaneps AJ, Geor RJ, Bayly W (2014). Veterinary aspects of training and competing polo ponies. Equine sports medicine and surgery.

[13] Marlin DJ, Allen JC (1999). Cardiovascular demands of competition on low-goal (non-elite) polo ponies. Equine Vet J.

[14] Lindholm A, Piehl K (1974). Fibre composition, enzyme activity and concentrations of metabolites and electrolytes in muscles of standardbred horses. Acta Vet Scand.

[15] Galisteo AM, Agiüera E, Monterde JG, Miró F (1992). Gluteus medius muscle fiber type composition in young Andalusian and Arabian horses. J Equine Vet Sci.

[16] Lopez-Rivero JL, Serrano AL, Diz AM, Galisteo AM. Variability of muscle fibre composition and fibre size in the horse gluteus medius: an enzyme-histochemical and morphometric study. J Anat. 1992;181( Pt 1):1-10. PMC1259747 1284127

[17] Ginneken MME (2006). Adaptation of signal transduction and muscle proteome in trained horses.

[18] Kawai M, Aida H, Hiraga A, Miyata H (2013). Muscle satellite cells are activated after exercise to exhaustion in Thoroughbred horses. Equine Vet J.

[19] Srikuea R, Symons TB, Long DE, Lee JD, Shang Y, Chomentowski PJ, Yu G, Crofford LJ, Peterson CA (2013). Association of fibromyalgia with altered skeletal muscle characteristics which may contribute to postexertional fatigue in postmenopausal women. Arthritis Rheum.

[20] Lillie RD, Ashburn LL (1943). Supersaturated solutions of fat stains in dilute isopropanol for demonstration of acute fatty degeneration not shown by Herxheimer’s technique. Arch Pathol.

[21] Kocsis T, Baan J, Muller G, Mendler L, Dux L, Keller-Pinter A (2014). Skeletal muscle cellularity and glycogen distribution in the hypermuscular Compact mice. Eur J Histochem.

[22] Rinnankoski-Tuikka R, Silvennoinen M, Torvinen S, Hulmi JJ, Lehti M, Kivela R, Reunanen H, Kainulainen H (2012). Effects of high-fat diet and physical activity on pyruvate dehydrogenase kinase-4 in mouse skeletal muscle. Nutr Metab (Lond).

[23] Thompson WR, Gordon NF, Pescatello LS, Medicine ACS (2010). Benefit and risks associated with physical activity. ACSM’s guidelines for exercise testing and prescription.

[24] Zobba R, Ardu M, Niccolini S, Cubeddu F, Dimauro C, Bonelli P, Dedola C, Visco S, Parpaglia MLP (2011). Physical, hematological, and biochemical responses to acute intense exercise in polo horses. J Equine Vet Sci.

[25] Spurway NC (1992). Aerobic exercise, anaerobic exercise and the lactate threshold. Br Med Bull.

[26] Murphy M, O’Connor L, Walsh D, Condon S (1985). Oxygen dependent lactate utilization by Lactobacillus plantarum. Arch Microbiol.

[27] Guy PS, Snow DH (1977). The effect of training and detraining on muscle composition in the horse. J Physiol.

[28] Serrano A, Quiroz-Rothe E, Rivero J-L (2000). Early and long-term changes of equine skeletal muscle in response to endurance training and detraining. Pflugers Arch.

[29] Brown MD, Hudlická O. Angiogenesis in Skeletal and Cardiac Muscle. In: Fan T-PD, Kohn EC, editors. The New Angiotherapy. Totowa, NJ: Humana Press; 2002. p. 213-48.

[30] Prior BM, Yang H, Terjung RL (2004). What makes vessels grow with exercise training?. J Appl Physiol.

