# Supplementary material for: Modulating effects of exercise training regimen on skeletal muscle properties in female polo ponies
Source: BMC Vet Res. 2016 Nov 4;12:245. doi: 10.1186/s12917-016-0874-6 (PMC5095958; doi:10.1186/s12917-016-0874-6)
Supplement: Additional file 1: Table S1. — Muscle fiber type I obtained after different periods of exercise training and activities. (DOCX 20 kb) [file 12917_2016_874_MOESM1_ESM.docx]

**Additional file 1**

**Modulating effects of exercise training regimen in polo ponies on skeletal muscle properties**

Metha Chanda, Ratchakrit Srikuea, Worakij Cherdchutam, Arthit Chairoungdua, and Pawinee Piyachaturawat

**Table S1.** Muscle fiber type I obtained after different periods of exercise training and activities.

| **Horse**  **number** | **Muscle fiber type I (%)** | | | |
| --- | --- | --- | --- | --- |
|  | **B** | **L** | **LM** | **LMP** |
| 1 | 15.87 | 15.67 | 13.76 | 21.75 |
| 2 | 20.82 | 21.80 | 18.14 | 17.44 |
| 3 | 20.14 | 26.09 | 27.34 | 35.09 |
| 4 | 21.91 | 16.55 | 20.29 | 28.22 |
| 5 | 38.09 | 35.91 | 35.39 | 30.86 |
| 6 | 22.07 | 25.80 | 27.45 | 24.87 |
| 7 | 38.83 | 34.66 | 34.74 | 46.01 |
| 8 | 24.76 | 25.84 | 38.43 | 27.94 |
| 9 | 23.02 | 23.86 | 20.62 | 33.17 |
| Mean±SD | 25.06±7.98 | 26.34±5.66 | 26.24±8.63 | 29.48±8.29 |

B= basal activity, L= low intensity exercise, LM= low to moderated intensity exercise, LMP= low to moderated intensity exercise plus match play**.**

**Table S2**: Muscle fiber type IIa obtained after different periods of exercise training and activities.

| **Horse**  **number** | **Muscle fiber type IIa (%)** | | | |
| --- | --- | --- | --- | --- |
|  | **B** | **L** | **LM** | **LMP** |
| 1 | 38.94 | 41.25 | 41.59 | 46.95 |
| 2 | 42.23 | 42.56 | 44.88 | 54.22 |
| 3 | 53.55 | 42.32 | 39.86 | 46.96 |
| 4 | 46.39 | 48.20 | 46.93 | 41.41 |
| 5 | 39.51 | 45.51 | 36.18 | 47.48 |
| 6 | 44.83 | 59.24 | 55.85 | 58.47 |
| 7 | 37.25 | 35.28 | 24.05 | 46.63 |
| 8 | 52.64 | 45.59 | 33.76 | 46.98 |
| 9 | 49.07 | 41.29 | 48.57 | 51.65 |
| Mean±SD | 44.93±5.97 | 44.10±4.72 | 44.30±5.77 | 48.97±5.02 |

B= basal activities, L= low intensity exercise, LM= low to moderated intensity exercise, LMP= low to moderated intensity exercise plus match play.

**Table S3**: Muscle fiber type IIa/x obtained after different periods of exercise training and activities.

| **Horse**  **number** | **Muscle fiber type IIa/x (%)** | | | |
| --- | --- | --- | --- | --- |
|  | **B** | **L** | **LM** | **LMP** |
| 1 | 6.73 | 8.63 | 5.50 | 6.63 |
| 2 | 6.74 | 4.57 | 6.51 | 7.63 |
| 3 | 7.35 | 7.34 | 4.78 | 9.23 |
| 4 | 7.93 | 7.08 | 6.76 | 10.74 |
| 5 | 2.04 | 4.82 | 5.37 | 5.93 |
| 6 | 7.82 | 6.34 | 4.30 | 6.88 |
| 7 | 2.48 | 5.97 | 11.36 | 1.84 |
| 8 | 3.37 | 5.54 | 6.79 | 8.89 |
| 9 | 7.67 | 8.58 | 6.16 | 5.82 |
| Mean±SD | 5.79±2.43 | 6.54±1.49 | 6.39±2.06 | 7.07±2.56 |

B= basal activity, L= low intensity exercise, LM= low to moderated intensity exercise, LMP= low to moderated intensity exercise plus match play.

**Table S4**: Muscle fiber type IIx obtained after different periods of exercise training and activities.

| **Horse number** | **Muscle fiber type IIx (%)** | | | |
| --- | --- | --- | --- | --- |
|  | **B** | **L** | **LM** | **LMP** |
| 1 | 38.46 | 31.25 | 30.14 | 24.67 |
| 2 | 30.26 | 27.71 | 28.47 | 20.71 |
| 3 | 18.96 | 24.01 | 26.02 | 8.71 |
| 4 | 23.78 | 25.07 | 25.02 | 19.63 |
| 5 | 20.37 | 23.80 | 23.06 | 15.73 |
| 6 | 25.29 | 21.20 | 20.41 | 9.79 |
| 7 | 21.44 | 20.45 | 22.84 | 5.52 |
| 8 | 19.23 | 21.41 | 21.02 | 16.19 |
| 9 | 25.23 | 26.27 | 24.64 | 9.37 |
| Mean±SD | 24.77±6.26 | 24.58±3.48 | 24.63±3.23 | 14.48±6.48 |

B= basal activities, L= low intensity exercise, LM= low to moderated intensity exercise, LMP= low to moderated intensity exercise plus match play.
